# We Might Have a Sports Gambling Problem and It Is Not What You Think: A Commentary

**DOI:** 10.1007/s10899-024-10315-5

**Published:** 2024-05-28

**Authors:** James P. Whelan, Rory A. Pfund, Meredith K. Ginley

**Affiliations:** 1 Tennessee Institute for Gambling Education & Research, The University of Memphis, Memphis, TN 38152 United States; 2 Department of Psychology, University of Memphis, Memphis, United States; 3 Department of Psychology, East Tennessee State University, Tennessee, United States

## Abstract

The 2018 Supreme Court decision on Murphy v. National Collegiate Athletic Association brought not only a change in the United States commercial gambling landscape, but also considerable speculation across public forums about whether expanded sports gambling causes new, distinct, and greater harm than existing legal forms of gambling. This commentary brings into the focus that the definition of this form of gambling has recently begun to shift without a theoretical basis or empirical evidence. To bring evidence to bear, there is a need for a precise operational definition of sport gambling and greatly clarity to the questions that can drive knowledge generation.

## Introduction

Questions and concerns about the impact of sports gambling have risen quickly following the expansion of legalization in the United States (Enrich, 2022; Miller, 2023). At the time of this court decision, the only legal venues for sportsbooks were in-person in Nevada, although three states did have limited sports gambling operations (Meer, 2011). While various legal forms of gambling were available in 48 of the 50 states in the United States, the expansion of in-person and mobile betting on sports greatly expanded accessibility to this popular wagering option. A common and reasonable theme across media platforms has been speculation about whether expanded sports gambling causes new, distinct, and/or greater harm than existing forms of gambling. The concerns are not without merit. As an example, the organization representing commercial and tribal gambling revenue in the United States (US) reported that $2.79 billion was wagered on sports in the US during the first quarter of 2023, an increase of over 70% from the previous year (American Gaming Association, 2023). Many stakeholders, including government regulators, have turned to the research community to inquire whether the ending costs associated with gambling harms will overwhelm us (Lehr, 2023).

The definition of sports gambling and the activities included under that definition have appeared consistent until recently. Kallick and colleagues (1979) published what might be the earliest survey of gambling in the US. They considered sports gambling as placing bets on traditional single game-sporting events (e.g., football, tennis, golf, etc.) and excluded wagering on animal racing. This same definition was subsequently adopted in the prevalence work by Rachel Volberg (e.g., 1996), including the important 1999 National Opinion Research Center study. Welte and colleagues in 2002 and 2015 prevalence studies also continued with the same definition of sports gambling, although online sports gambling was treated as a separate activity.

More recently, this definition has begun to evolve. The rise in popularity of fantasy sports gambling brought with it questions about whether it constitutes a form of gambling (Derevensky & Marchica, 2018). However, again, this activity was treated as separate from “traditional” or general sports gambling in a survey project by the National Council on Problem Gambling (2019) and a systematic review by Winters and Derevensky (2020). Similarly, esports betting has entered the conversation. Legal and practical parallels between sports gambling and e-sports gambling (e.g., Macey et al., 2021; Sweeney et al., 2021) have been made without anyone providing a conceptual or empirical argument that esports gambling is a form of sports gambling. Recent publications have defined sports gambling as the combination of general sports gambling, paid fantasy league play, daily fantasy league play, and e-sports gambling (e.g., Grubbs & Kraus, 2022, Grubbs & Kraus, 2023). Interestingly, this paper excluded in-play bets that involve proposition bets and micro-bets that are essentially side bets indirectly linked or not at all linked to the game outcome. For many, the opportunity for in-game bets is a key component of betting on sports that is made possible by the dynamic live odds not available during other types of gambling, such as horse racing (Killick & Griffiths, 2019).

A clear and precise definition of sports gambling is necessary for researchers to best communicate with media, the public, and policy makers. Is a sports gambling category unified in that all behaviors involve betting on a competition? If so, should bets on a horse racing be considered sports gambling? Or perhaps is the real question about ease of access to wagering? Winters and Derevensky (2020) highlighted the role of online access and the general popularity of sports in their review. A definition lacking specific details about what constitutes sports gambling, or more importantly, what about sports gambling makes it distinct from other forms of gambling, clouds the interpretation of results about what the public, media, individuals, and other parties perceive as sports gambling versus not.

Beyond the call for a research-based definition of gambling (Williams et al., 2017), methodological and definitional questions about sports gambling are critical for creating a foundation of research on this form of gambling and, importantly, associated harms. Perhaps, as implied by recent publications about sports gambling, there is a gambling taxonomy, which would need to include rules for linking current and future forms of gambling. Such a taxonomy might suggest rules for grouping, separating, and linking different gambling forms because of the characteristics of the wagering, the level of knowledge or skill that would provide greater or worse odds to the person wagering, or perhaps the ease of access to the opportunity to wager. This taxonomy could potentially inform a quantitative estimate of the risk associated with various types of bets or a specific bet (e.g., Edson et al., 2023) that could be akin to a standard drink/dose calculation.

Alternatively, empirical efforts might strive to identify the key elements of the gambling process that are central to prevention, assessment, and treatment of gambling harm, problem gambling, and gambling disorder. Perhaps, research to identify the process of gambling that enhances the likelihood of gambling with consideration of risk or provides the individual with confidence that they have an advantage. The field must strive for evidence to help formulate the most critical questions and prioritize them to best understand sports gambling and its associated harms.

As these critical empirical questions are addressed with prospective data, a standard definition of sports gambling is needed. For now, we propose that the field should continue using the historical definition of sports gambling to remain consistent in the long-held standard of the literature: wagering on traditional single-game sports competitions, and on the elements within those sports competitions. We suggest that both rational and empirical efforts guide the modernization and expansion of this definition to include newer forms of gambling (e.g., paid fantasy league play, daily fantasy league play, and e-sports gambling). Beyond this definition clarity, we wonder about the meaningfulness of asking questions about whether distinct forms of gambling result in unique harms. We hypothesize that as with electronic gambling machines gambling harm is more about the dose and preference than the gambling type (e.g., Dowling et al., 2005).
